# Optimizing Nystatin Biosynthesis Through Targeted Manipulation of *nysL* and *nysGtf* in *Streptomyces noursei*

**DOI:** 10.3390/ijms27219572

**Published:** 2026-10-26

**Authors:** Jibin Shi, Dexi Song, Junran Yang, Xianpu Ni, Yitong Xu, Fuju Wang, Kristiina Ylihonko, Mikko Metsä-Ketelä, Ming Song, Weizhuo Xu

**Affiliations:** 1 School of Life Sciences and Biopharmaceuticals, Shenyang Pharmaceutical University, 103 Wenhua Road, Shenhe District, Shenyang 110016, China; 2 School of Functional Food and Wine, Shenyang Pharmaceutical University, 103 Wenhua Road, Shenhe District, Shenyang 110016, China; 3 School of Traditional Chinese Materia Medica, Shenyang Pharmaceutical University, 103 Wenhua Road, Shenhe District, Shenyang 110016, China; 4 Beijing Global Biologicals Inc., 99 Yuexiu Road, Beijing 100192, China; 5 Care4living Oy, Itäinen Pitkäkatu 4, 20520 Turku, Finland; 6 Department of Life Technologies, University of Turku, Tykistökatu 6, 20520 Turku, Finland

**Keywords:** *Streptomyces noursei*, biosynthesis, *nysL* gene, *nysGtf* gene, P450 monooxygenase, glycosyltransferase

## Abstract

Nystatin, a polyene macrolide antifungal agent produced by *Streptomyces noursei*, exists as three major derivatives (nystatin A1, A3, polyfungin B) with distinct C-10 and C-35 modifications. Although the core catalytic functions of NysL and NysGtf have been established, the metabolic crosstalk between the C-10 hydroxylation and C-35 digitosylation branches, together with the in vivo substrate promiscuity of NysGtf in industrial *S. noursei*, remains poorly understood. In this study, we constructed gene-disruption, complementation and overexpression mutants of *S. noursei* 72-22-1 to characterize *nysL* and *nysGtf*. While NysL has previously been established as the C-10 hydroxylase, we characterized its role in the industrial derivative strain 72-22-1. *nysL* disruption abolished C-10 hydroxylation, eliminated nystatin A1/A3 and caused accumulation of 10-deoxynystatin and polyfungin B, while complementation and/or overexpression restored A1/A3 formation. *nysGtf* inactivation blocked C-35 glycosylation, raising nystatin A1 titer to 0.587 mg/mL and decreasing polyfungin B to 0.027 mg/mL. Its overexpression enhanced nystatin A3 production to 1.195 mg/mL. Our findings define the physiological roles of NysL and NysGtf in nystatin biosynthesis, identify polyfungin B as the key antifungal component, and provide a feasible strategy for metabolic engineering of *S. noursei* to generate high-activity nystatin derivatives.

## 1. Introduction

**Figure 1:**
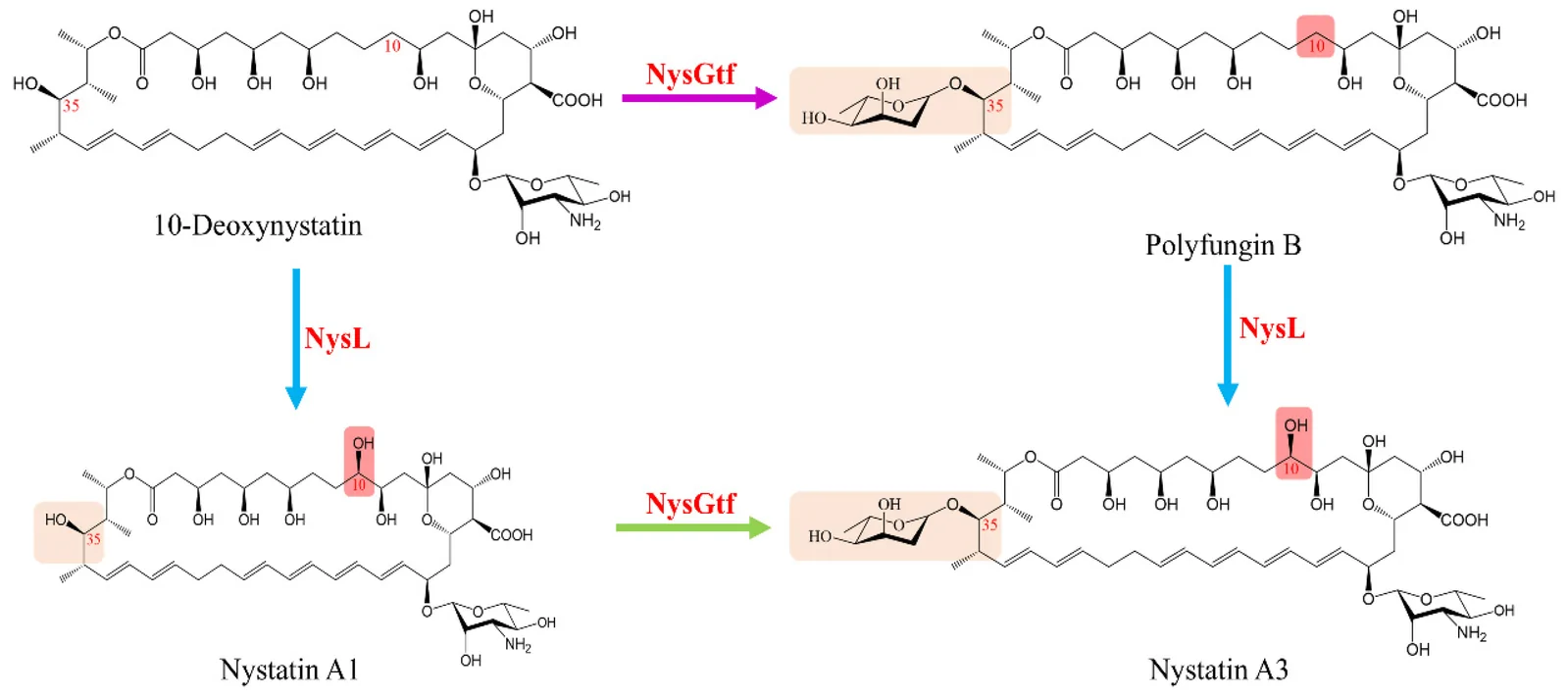
The role of NysL and NysGtf in the synthesis of nystatin A1, nystatin A3 and polyfungin B.

Fungal infections pose a global health threat, ranging from superficial rashes to life-threatening systemic diseases. Invasive candidiasis and aspergillosis exhibit mortality rates exceeding those of bacterial infections, particularly in immunocompromised individuals and intensive care unit (ICU) patients [1,2]. Nystatin, also known as fungicidin or mycostatin, is a polyene macrolide antibiotic. Nystatin was first isolated from *Streptomyces noursei* culture by Hazen and Brown in 1950 [3]. Its main components are nystatin A1, nystatin A3, and polyfungin B [4,5]. These compounds share an identical structural framework, with primary distinctions residing in the hydroxyl group at the C-10 position and the digitoxose sugar moiety at the C-35 position. Nystatin A1 and A3 possess an additional hydroxyl group at C-10 compared to polyfungin B. In contrast to nystatin A1, nystatin A3 and polyfungin B are characterized by an extra digitoxose sugar at C-35 (Figure 1). Notably, under equal mass conditions, polyfungin B exhibits significantly higher antifungal activity than nystatin A1 and A3 [6]. Therefore, investigating the biosynthetic pathway of polyfungin B holds substantial research value and lays the groundwork for future enhancement of its production through genetic engineering approaches.

*Streptomyces*, as filamentous Gram-positive bacteria, could produce diverse secondary metabolites with distinct chemical properties and bioactivities [7,8,9]. Zotchev et al. identified the biosynthetic gene cluster for nystatin A1 in *Streptomyces noursei* ATCC 11455, which comprises six polyketide synthase (PKS) genes (*nysA*, *nysB*, *nysC*, *nysI*, *nysJ*, and *nysK*), collectively encoding 19 modules of a modular polyketide synthase [10]. The biosynthetic pathways of many antibiotics produced by *Streptomyces* involve steps catalyzed by P450 monooxygenases, which are primarily responsible for the hydroxylation of intermediates in the synthetic pathway [11,12]. As previously demonstrated in *S. noursei* ATCC 11455 [12], the key C-10 hydroxylation modification of nystatin A1 is catalyzed by P450 monooxygenase NysL, which converts 10-deoxynystatin into nystatin A1 (Figure 1). However, comprehensive quantitative profiling of how *nysL* perturbation reshapes the full panel of nystatin derivatives (including polyfungin B and 10-deoxynystatin) has not been reported, especially for the industrially relevant mutant strain 72-22-1. The functional significance of C-10 hydroxylation for nystatin A1 antifungal activity is debated. While some studies suggest this modification is essential for active transport mechanisms [13], others demonstrate that 10-deoxynystatin exhibits comparable efficacy to nystatin A1 [12]. Despite unresolved mechanistic questions, NysL, the P450 monooxygenase catalyzing C-10 hydroxylation, represents a promising target for biosynthetic engineering. As the terminal step in nystatin A1 biosynthesis, rational modulation of NysL’s regioselectivity or epoxidation activity could enhance therapeutic properties without compromising downstream modifications. This strategy is supported by structural insights into the NysL catalytic mechanism and successful precedents in analogous P450 systems, including AmphL and PimD, which have yielded derivatives with improved efficacy [11].

Genes responsible for the synthesis and addition of GDP-mycosamine are found in the biosynthetic gene clusters of various polyene macrolide antibiotics, including candidin [14,15], amphotericin [16], nystatin [10], and pimaricin [17]. At present, it is generally believed that GDP-mycosamine is derived from the primary metabolite GDP-D-mannose via sequential enzymatic modifications. GDP-D-mannose is first converted to GDP-4-keto-6-deoxy-D-mannose by 4,6-dehydratases (NysDIII, AmphDIII, PimJ, FscMIII), and then isomerized to GDP-3-keto-6-deoxy-D-mannose at positions 3 and 4, which is further converted to GDP-mycosamine by transaminases (NysDII, AmphDII, PimC, FscMII). The activated glycosyl donor is subsequently recognized by glycosyltransferases (NysDI, AmphDI, PimK, FscMI), which transfer the sugar moiety to specific positions on the macrolactone aglycone. This final glycosylation step is essential for conferring bioactivity to polyene macrolide antibiotics [17,18]. Mirza et al. have established the core mechanism of C-35 L-digitoxose transfer catalysed by SnNysSV [19]. Nevertheless, the substrate promiscuity of this glycosyltransferase variant and the resulting metabolic flux redistribution in the industrial *S. noursei* strain remain to be explored. The main structural differences among the three components of nystatin A1, nystatin A3, and polyfungin B lie in the hydroxyl group at the C10 position and the digoxin sugar at the C35 position. However, the biosynthetic pathway of the digitoxose sugar in nystatin is still unknown. Bihlmaier et al. reported the biosynthesis and linkage mechanisms of digitoxose in α-lipomycin [20]. Their findings provide critical insights for elucidating the C-35 digitoxose biosynthesis and glycosylation processes in nystatin A3 and polyfungin B, offering substantial inspiration for this study.

There is currently no literature reporting the detailed biosynthetic pathways of nystatin A3 and polyfungin B. Therefore, studying the synthesis and related gene functions of nystatin A3 and Polyfungin B is of great significance. The parental strain used in this study, *Streptomyces noursei* 72-22-1, was derived from *Streptomyces noursei* D-3-14 via UV mutagenesis [4]. Genomic sequencing confirmed that its genome contains a complete biosynthetic gene cluster of nystatin. Bioinformatics analysis identified gene 3-545 as *nysL*, encoding a P450 monooxygenase responsible for C-10 hydroxylation, and gene 3-1219 as *nysGtf*, putatively responsible for attachment of the digitoxose moiety at C-35. Furthermore, homology analysis revealed no significant sequence similarity between NysDI and NysGtf, supporting the functional distinction between these two enzymes in the nystatin biosynthetic pathway. This study pioneers a combinatorial metabolic engineering approach to optimize nystatin biosynthesis in *Streptomyces noursei*. We demonstrate the synergistic regulation of *nysL* (P450 hydroxylase) and *nysGtf* (glycosyltransferase) significantly improves pathway efficiency. Second, we achieve the isolation and structural characterization of the critical intermediate 10-deoxynystatin, previously inaccessible in this strain, through NMR (Nuclear Magnetic Resonance) and LC-MS (Liquid Chromatography-Mass Spectrometry) analysis.

## 2. Results

### 2.1. Construction of the nysL and nysGtf Gene Disruption Strains

**Figure 2:**
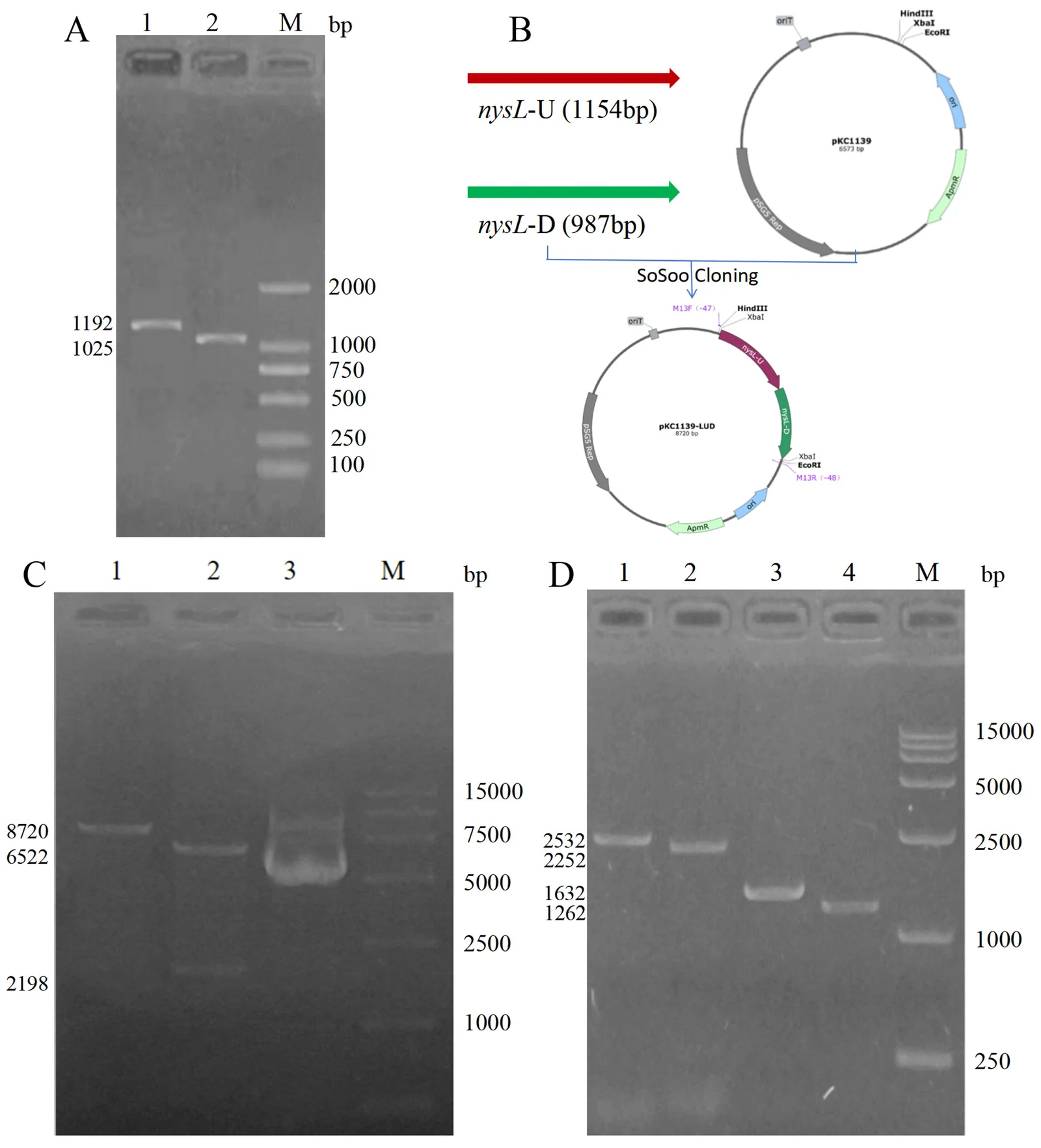
Construction of the nysL Gene Disruption Strain. (**A**). PCR products of upstream and downstream homologous arms (*nysL*−U 1192 bp and *nysL*−D 1025 bp). (**B**). Workflow for constructing pKC1139−LUD. (**C**). Restriction enzyme analysis of pKC1139−LUD. M: DL15000 DNA Marker; Lane1: pKC1139−LUD (Hind III); Lane2: pKC1139−LUD (Hind III/EcoR I); Lane3: pKC1139−LUD. (**D**). PCR product analysis of the *nysL* disruption strain. Lane1 PCR product (2532 bp) using upstream verification primers for the starting strain; Lane2. PCR product (2252 bp) using downstream verification primers for the starting strain; Lane3. PCR product (1632 bp) using upstream verification primers for the double-crossover strain; Lane4. PCR product (1262 bp) using downstream verification primers for the double-crossover strain; M: DL15,000 DNA Marker.

Plasmid pKC1139-LUD was introduced into *S. noursei* 72-22-1 by conjugation using *E. coli* ET12567 (pUZ8002, pKC1139-LUD), the *S. noursei ΔnysL* was successfully constructed. The *nysL*/*nysGtf* knockout mutants were verified by PCR using primers targeting the homologous recombination sites, and confirmed by Sanger sequencing of the PCR products (Figure 2A–D). Using the same method, plasmid pKC1139-GUD was introduced into *S. noursei* 72-22-1 by conjugation using E. coli ET12567 (pUZ8002, pKC1139-GUD), *S. noursei ΔnysGtf* was successfully constructed (Figure S1A–C).

### 2.2. Analysis of Fermentation Products in the nysL Disruption, Complementation and Overexpression Strains

**Figure 3:**
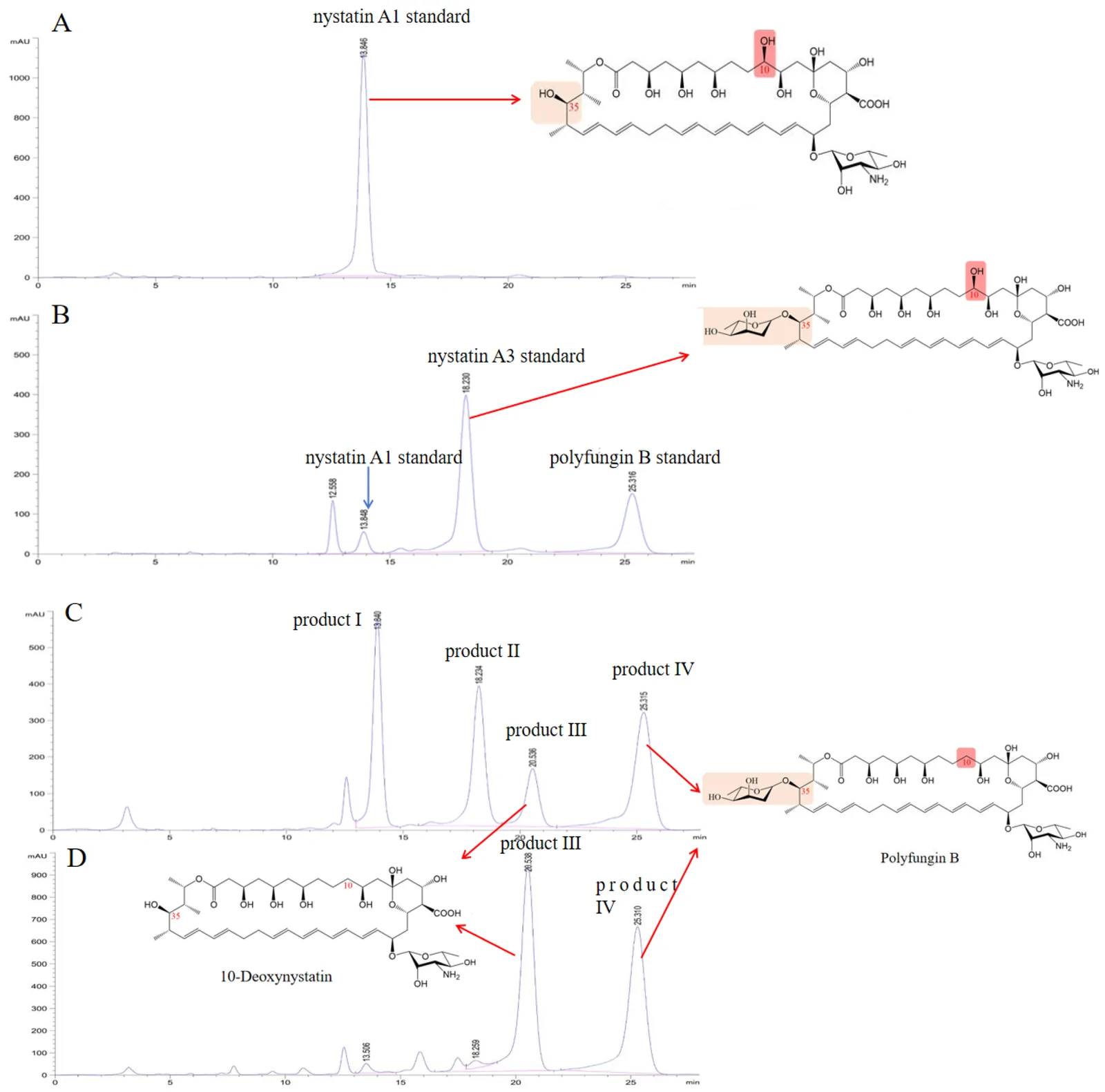
HPLC analysis of fermentation products in *S. noursei ΔnysL* and *S. noursei* 72-22-1 (**A**). nystatin A1 standard (**B**). Nystatin standard (**C**). Fermentation products in *S. noursei* 72-22-1 (**D**). Fermentation products in *S. noursei ΔnysL.* I: nystatin A1, II: nystatin A3, III: 10-deoxynystatin, IV: polyfungin B.

The HPLC analysis results of nystatin standard (nystatin A1, nystatin A3, and polyfungin B) shown in Figure 3A,B. Comparative analysis of fermentation products from the *nysL* disruption strain (*S. noursei ΔnysL*) and the starting strain (*S. noursei* 72-22-1) revealed differences in the production profiles. In *S. noursei* 72-22-1, four fermentation products I, II, III, and IV were observed (Figure 3C). In contrast, *S. noursei ΔnysL* produced only two major products that corresponded to III and IV (Figure 3D). Analysis of the fermentation products of *S. noursei* 72-22-1 by HPLC suggested that products I, II, and IV are nystatin A1, nystatin A3, and polyfungin B, respectively. In the fermentation products of *S. noursei ΔnysL*, product IV was identified as polyfungin B (Figure 3D). Product III from *S. noursei* 72-22-1 and III from *S. noursei ΔnysL* shared similar retention times, indicating they are the same substance. Based on the reported nystatin biosynthesis pathway and LC-MS analysis (Figure S2), this compound was inferred to be 10-deoxynystatin, a precursor of nystatin A1 [11].

**Figure 4:**
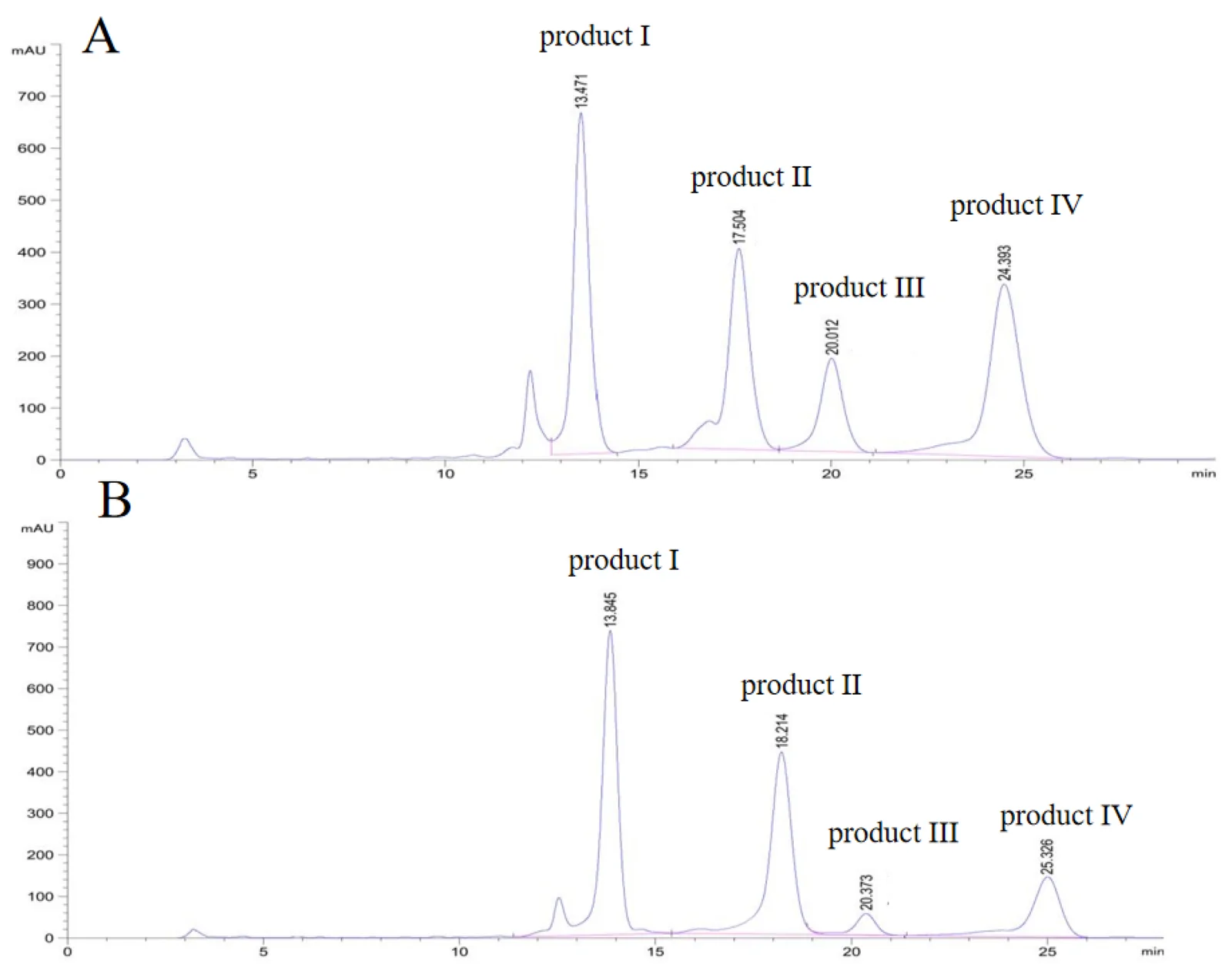
HPLC analysis of fermentation products in *S.noursei ΔnysL::nysL* (**A**) and *S. noursei::nysL* (**B**). Product I: nystatin A1; product II: nystatin A3; Product III: 10-deoxynystatin; Product IV: polyfungin B. The corresponding control samples are presented in Figure 3.

The sequence verified *nysL* complementation plasmid pSET152-nysL was introduced into *Streptomyces noursei ΔnysL* via conjugation. The complemented strain was designated *Streptomyces noursei ΔnysL::nysL*. HPLC analysis of the fermentation products revealed four major components I, II, III, IV (Figure 4A). These were identified as nystatin A1, nystatin A3, 10-deoxynystatin, and polyfungin B, respectively. The complemented strain restored the production of nystatin A1 and nystatin A3, while the accumulation of 10-deoxynystatin and polyfungin B was significantly reduced compared with the *ΔnysL* mutant. The restoration of the metabolite profile upon genetic complementation confirms that the altered metabolite phenotype observed in the *ΔnysL* mutant was caused by disruption of *nysL*.

The plasmid pSET152-nysL was also introduced into *S. noursei* 72-22-1 via conjugation transfer using *E. coli* ET12567 (pUZ8002, pSET152-nysL) as the donor strain [21,22]. The resulting overexpression strain was designated *S. noursei::nysL*. HPLC analysis of the fermentation products from *S. noursei::nysL* revealed three major products with retention times corresponding to nystatin A1 (I), nystatin A3 (II), and polyfungin B (IV), as well as a minor product tentatively identified as 10-deoxynystatin (III), the precursor of nystatin A1 (Figure 4B). This profile mirrored that of *S. noursei* 72-22-1.

### 2.3. The Effect of the nysL Gene on the Composition of Nystatin Derivatives

**Figure 5:**
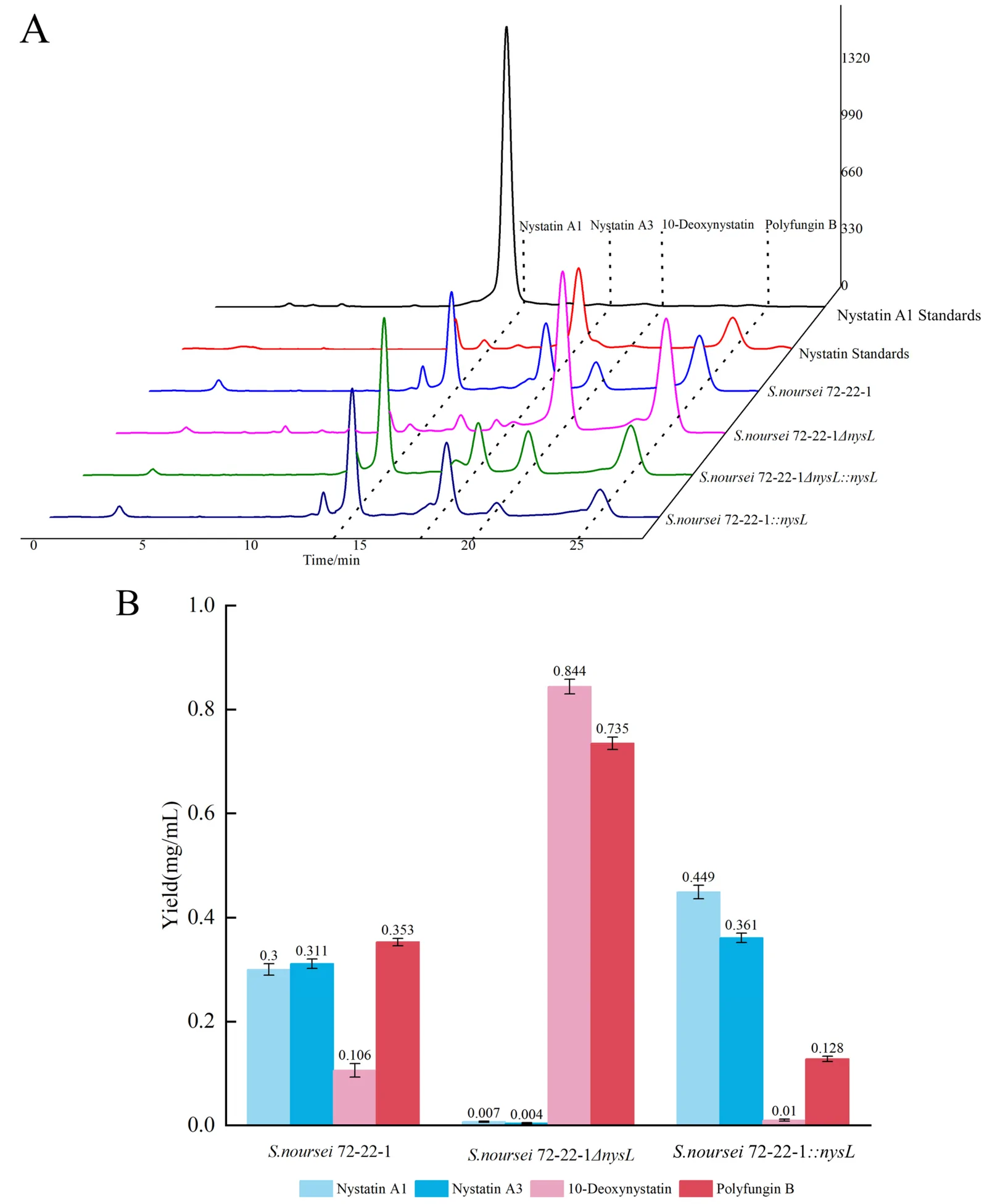
HPLC profiles and yields of nystatin derivatives in *S. noursei* 72-22-1 and nysL-engineered strains. (**A**). HPLC for different strains. (**B**). Impact of *nysL* on the yields of nystatin derivatives in *S. noursei* 72-22-1, *S. noursei* 72-22-1 *ΔnysL* and *S. noursei* 72-22-1*::nysL*.

The fermentation products of these engineered strains were subjected to HPLC analysis and yield quantification, as shown in Figure 5. In the fermentation broth of the starting strain *S. noursei* 72-22-1, the main products were identified as nystatin A1, nystatin A3, and polyfungin B. In contrast, the *nysL* knockout strain (*ΔnysL*) produced almost no detectable nystatin A1 or A3, and a new compound emerged in the chromatogram (Figure 5A). The newly appeared compound was identified as 10-deoxynystatin, a biosynthetic precursor of nystatin A1. The 10-deoxynystatin yield increased significantly from 0.106 mg/mL to 0.844 mg/mL (*p* < 0.01). The disruption of the *nysL* gene effectively reduced the production of nystatin A1 and nystatin A3 (Figure 5B). These results lay a foundation for the future construction of polyfungin B high-yield strains.

### 2.4. Structural Elucidation of Fermentation Products via MS and NMR Spectroscopy

To further confirm the chemical structures of products I, II, and IV, semi-preparative high-performance liquid chromatography (semi-preparative HPLC) was used for their isolation and purification, followed by both MS and NMR analyses for structural elucidation. Product III was characterized only by MS due to insufficient sample quantity for NMR measurements. The molecular formula of nystatin A1 is C_47_H_75_NO_17_, with a theoretical molecular weight of 925.62. Based on the mass spectrum analysis of product I, the observed MS (ESI) signals at *m*/*z* 926.62 [M+H]^+^ and *m*/*z* 948.61 [M+Na]^+^ indicate that product I corresponds to nystatin A1. Subsequently, ^13^C NMR spectroscopy was performed for further structural elucidation, and the results are summarized in Table 1. The chemical shifts at 176.19 ppm and 170.91 ppm correspond to two -C=O signals, while the peaks at 134.73 ppm, 131.72 ppm, 133.4 ppm (×3), 129.92 ppm, 131.6 ppm, 131.55 ppm, 134.73 ppm, 131.47 ppm, 129.71 ppm, and 135.9 ppm indicate the presence of six -C=C- bonds. Additionally, the peaks at 97.64 ppm and 97.82 ppm correspond to two -O-C-O- signals, whereas the signal at 56.38 ppm is attributed to a -CN- group.

The molecular formula of nystatin A3 is C_53_H_85_NO_20_, with a theoretical molecular weight of 1055.57. Based on MS (ESI) data, the characteristic ion peaks of product II were observed at *m*/*z* 1078.56 [M+Na]^+^, 1056.57 [M+H]^+^, 1038.57 [M-H_2_O+H]^+^, 1054.56 [M-H]^−^, and 1036.55 [M-H_2_O-H]^−^. The calculated molecular mass of product II was determined to be 1055.57, which is consistent with the relative molecular mass of nystatin A3 (C_53_H_85_NO_20_, 1056.25). Subsequently, ^1^H NMR analysis (600 MHz, DMSO-d_6_) of product II revealed resonance signals within the range of 5.5-6.22 ppm, which correspond to the six alkenyl proton signals common to nystatin A1, nystatin A3, and polyfungin B. Additionally, two methylene dioxy proton signals were observed at 4.72 and 4.78 ppm, demonstrating the presence of two glycosyl groups in product II. The ^13^C NMR spectrum (151 MHz, DMSO-d_6_) of product II exhibited a lactone carbon signal at 170.36 ppm and a carboxyl carbon at 174.27 ppm. Furthermore, hemiketal and hemiacetal carbon signals were identified at 97.31 and 97.83 ppm, respectively (Table 2).

The molecular formula of 10-deoxynystatin is C_47_H_75_NO_16_, serving as the precursor of nystatin A1, with a theoretical molecular weight of 910.09. Based on the HPLC-MS analysis of product III (Figure S2), MS(ESI)*m*/*z*[M+Na] + is 932.60, MS(ESI)*m*/*z*[M-H]^−^ is 908.77, the deductive molecular mass of product III is 909.69, corresponds to a molecular mass of 910.09, which aligns with the theoretical value of 10-deoxynystatin, confirming its identity. However, due to insufficient quantities of product III, further structural validation via NMR spectroscopy was not performed. The molecular formula of polyfungin B is C_53_H_85_NO_19_, with a theoretical molecular weight of 1040.25. The characteristic ion peaks of product IV were observed at *m*/*z* 1062.56 [M+Na]^+^, 1040.58 [M+H]^+^, 1022.57 [M-H_2_O+H]^+^, and 1038.56 [M-H]^−^, leading to the calculated molecular mass of product IV as 1039.57, which corresponds to the relative molecular mass of polyfungin B (C_53_H_85_NO_19_). The NMR spectra of product IV were largely similar to those of product II, with the primary distinction being the appearance of a signal at 38.24 ppm in the ^13^C NMR spectrum. Additionally, C-8 and C-12 exhibited downfield shifts, while C-9 showed an upfield shift, indicating the absence of a hydroxyl group at the C-10 position (Table 3).

### 2.5. The Effect of the nysGtf Gene on the Composition of Nystatin Derivatives

**Figure 6:**
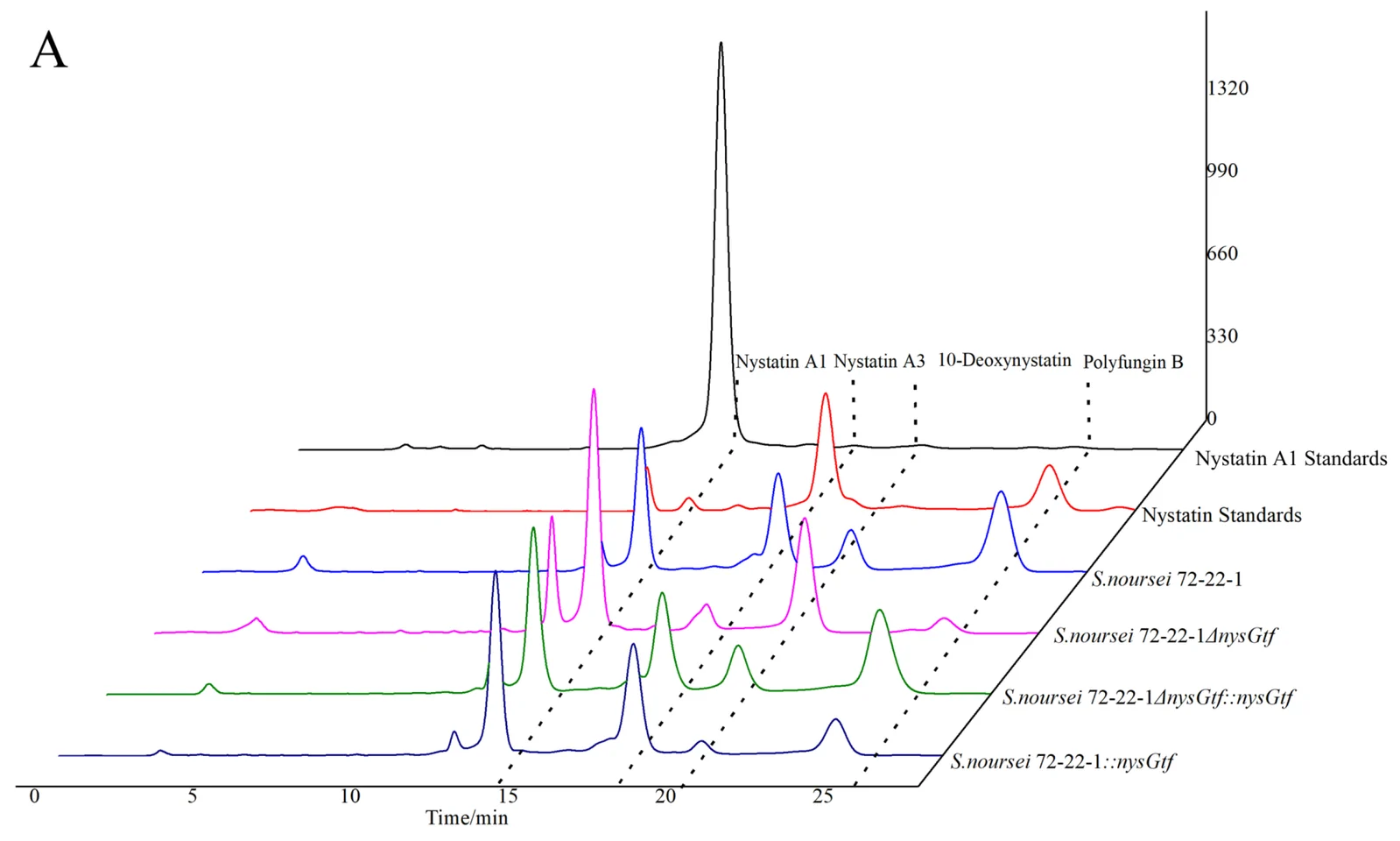

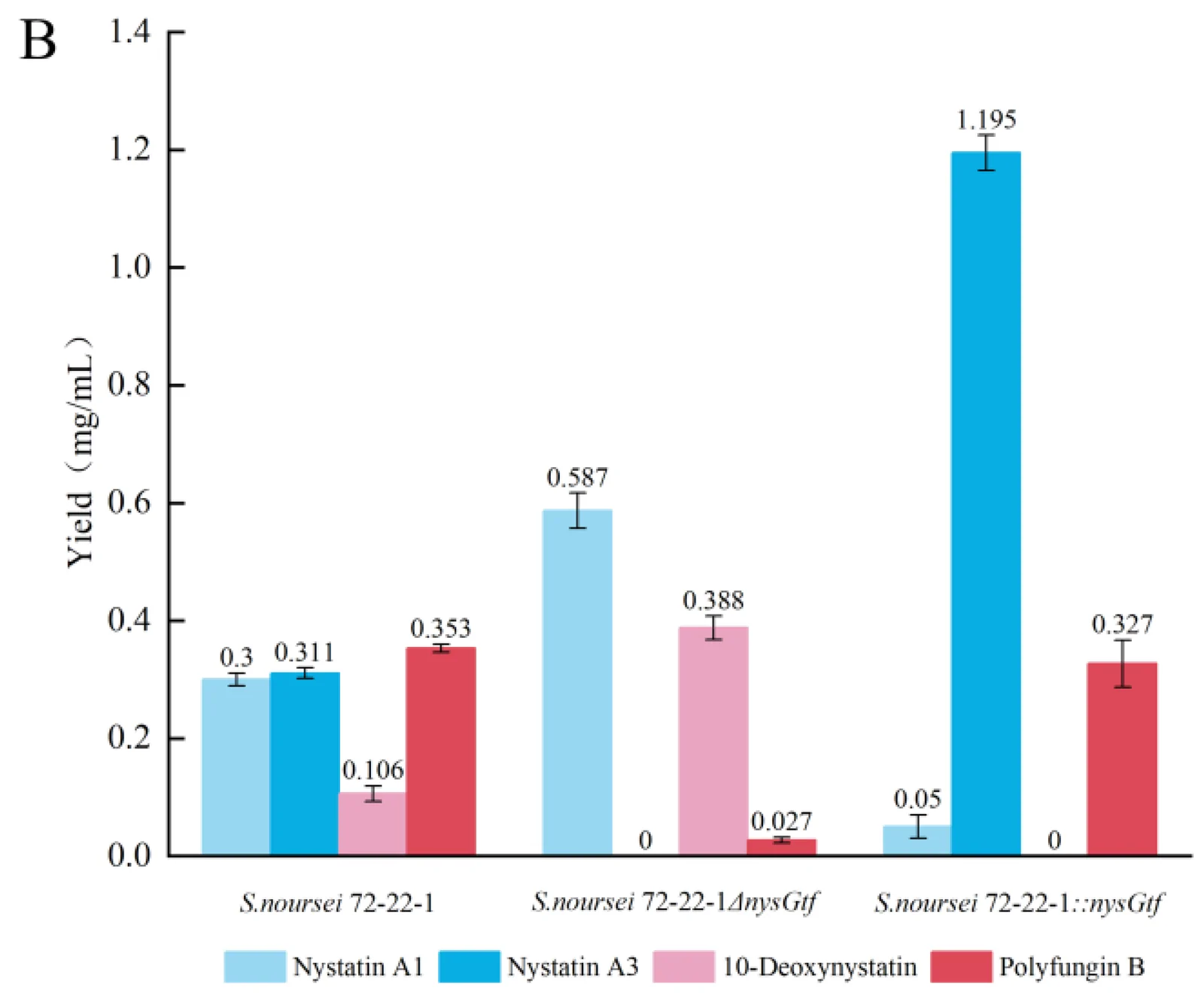
(**A**). HPLC for different strains. (**B**). Impact of *nysGtf* on the yields of nystatin derivatives in *S. noursei* 72-22-1, *S. noursei* 72-22-1*ΔnysGtf* and *S. noursei* 72-22-1*::nysGtf*.

Functional annotation of the *S. noursei* 72-22-1 genome using the antiSMASH online tool revealed a gene encoding a putative glycosyltransferase, which was subsequently designated as *nysGtf*. To verify the function of the *nysGtf* gene in *S. noursei* 72-22-1, the parental strain *S. noursei* 72-22-1 was used to construct a *nysGtf* knockout mutant (*S. noursei* 72-22-1*ΔnysGtf*), a complementation strain (*S. noursei* 72-22-1*ΔnysGtf::nysGtf*), and an overexpression strain (*S. noursei* 72-22-1*::nysGtf*) via conjugation-based gene transfer. The fermentation products of these engineered strains were analyzed by HPLC to assess compound composition and yield, as shown in Figure 6. It was observed that deletion of the *nysGtf* gene abolished the production of nystatin A3. Meanwhile, the yield of nystatin A1 increased from 0.3 mg/mL to 0.587 mg/mL (*p* < 0.01), and the yield of 10-deoxynystatin rose from 0.106 mg/mL to 0.388 mg/mL (*p* < 0.01). In contrast, the production of polyfungin B decreased significantly from 0.353 mg/mL to 0.027 mg/mL (*p* < 0.01). These results suggest that knockout of *nysGtf* effectively blocks C-35 glycosylation, thereby preventing the biosynthesis of both nystatin A3 and polyfungin B. However, since the production of polyfungin B did not drop to zero, it is speculated that an alternative compensatory pathway may exist in the genome, allowing for trace-level biosynthesis of polyfungin B. To further validate the function of the *nysGtf* gene, complementation and overexpression experiments were conducted. In the overexpression strain *S. noursei* 72-22-1::*nysGtf*, the yield of nystatin A3 increased significantly from 0.311 mg/mL to 1.195 mg/mL (*p* < 0.01), confirming that the glycosyltransferase encoded by *nysGtf* is responsible for C-35 digitoxose glycosylation.

## 3. Discussion

Glycosyltransferases in *Streptomyces* primarily mediate glycosylation during the late-stage modification of antibiotics. These enzymes utilize NDP-sugars to transfer sugar moieties to specific O-, N-, or even C-atoms of aglycone scaffolds. The attached sugars are typically deoxysugars uncommon in primary metabolism, often modified with amino or methyl groups [23,24,25]. Most glycosylated polyene macrolides, such as nystatin, are decorated with D-mycosamine, a 6-deoxyaminosugar, which confers specificity for binding ergosterol in fungal cell membranes [26]. The functional validation of NysGtf establishes a paradigm for dissecting C-hydroxylation and deoxysugar transferase specificity in polyene pathways, which are often conserved across *Streptomyces* species. This research can guide future studies on other clinically relevant polyenes (amphotericin B, pimaricin), where glycosyltransferase function remained uncharacterized.

Gene disruption remains a cornerstone methodology for elucidating the precise physiological roles of biosynthetic enzymes within complex secondary metabolic pathways [27]. Compared to heterologous expression and transcriptomic profiling, gene disruption offers direct in vivo evidence for enzymatic function by unveiling metabolic phenotypes, a strategy validated in *Streptomyces* metabolite biosynthesis [14,28,29]. Our targeted disruption of *nysL* and *nysGtf* in *S. noursei* 72-22-1 unequivocally demonstrates the indispensable functions of NysL in C-10 hydroxylation and NysGtf in C-35 glycosylation during nystatin biosynthesis. The C-10 hydroxylase activity of NysL was originally demonstrated in the type strain *S. noursei* ATCC 11455 by Volokhan et al. [12]. Our present work confirms this function in the UV-mutagened industrial strain *S. noursei* 72-22-1 and further provides full quantitative metabolite datasets for all major nystatin-related compounds upon *nysL* disruption, complementation and overexpression. Moreover, we successfully accumulated and structurally characterized the intermediate 10-deoxynystatin in this strain background, and could interpret the metabolic branching together with the newly characterized glycosyltransferase NysGtf. The accumulation of pathway intermediates, 10-deoxynystatin and polyfungin B in *S. noursei ΔnysL*, and nystatin A1 and 10-deoxynystatin in *S. noursei ΔnysGtf*, provides definitive in vivo evidence of enzymatic blockades at these specific modification steps. This genetic dissection approach resolves long-standing ambiguities regarding the catalytic specificity of these enzymes within the nystatin pathway, particularly for NysGtf, whose digitoxose-transferase activity had not been experimentally validated prior to this study. The success of disrupting *nysL* and *nysGtf* in *S. noursei* highlights the utility of genetic dissection in untangling multi-step modification pathways. This approach is particularly valuable for polyene macrolides, where overlapping intermediates and promiscuous enzymes often obscure individual enzyme contributions. The functional validation through complementation experiments further solidifies these assignments. The restoration of nystatin A1/A3 production in *S. noursei ΔnysL::nysL* and nystatin A3 in *S. noursei ΔnysGtf::nysGtf* confirms that the observed metabolic phenotypes directly result from the targeted gene disruptions. Critically, these findings extend beyond confirming enzyme activities; they reveal the metabolic plasticity of the pathway. For instance, the 284.2% increase in nystatin A3 yield upon *nysGtf* overexpression demonstrates how functional characterization enables rational pathway engineering-redirecting metabolic flux toward desired derivatives by manipulating validated genetic targets. This principle is further evidenced by the 83.3% reduction in nystatin A1 accumulation in the overexpression strain, highlighting the function of NysGtf in glycosylating. The results demonstrate that targeted manipulation of validated functional genes is more efficient than random mutagenesis for strain improvement. This precision reduces off-target metabolic burden and accelerates industrialization [30,31]. In addition, the accumulation of 10-deoxynystatin and polyfungin B in disruption strains provides a biological source for these rare intermediates, which were previously difficult to obtain via chemical synthesis. Such compounds can serve as lead molecules for semi-synthetic modification. For instance, further chemical or biological modification of 10-deoxynystatin may reduce host toxicity while retaining antifungal activity, thereby addressing a critical limitation of current polyene therapeutics.

While prior studies had implicated P450 monooxygenases in polyene hydroxylation [10,11], our work provides comprehensive quantification of pathway flux alterations and establishes the function of NysGtf. This mechanistic clarity is fundamental for future applications: engineering regioselectivity in NysL homologs or exploiting substrate flexibility of NysGtf could generate novel antifungal derivatives. The *nysGtf* gene specifically regulates the synthesis ratio of nystatin derivatives by modulating the C-35 glycosylation step, with the following key conclusions: (1) *nysGtf* is an essential gene for nystatin A3 and polyfungin B biosynthesis. Disruption of *nysGtf* led to an almost complete loss of nystatin A3 and a significant reduction in polyfungin B production, while complementation of *nysGtf* restored their yields to starting strain levels. These results confirm that *nysGtf* encodes a glycosyltransferase responsible for C-35 digitoxose glycosylation, which is indispensable for the biosynthesis of both A3 and polyfungin B. *nysGtf* encodes a glycosyltransferase homologous to SnNysSV reported by Mirza et al. [19], with 96.96% amino acid identity with SnNysSV (Table S3). (2) *nysGtf* exhibits stronger regulatory specificity for nystatin A3 synthesis. Overexpression of *nysGtf* resulted in a marked increase in nystatin A3 yield, whereas polyfungin B production only slightly increased and did not exceed the starting strain level. This suggests that *nysGtf* has a higher substrate preference for the A3 precursor, leading to preferential channeling of metabolic flux toward A3 biosynthesis when the enzyme is overexpressed. (3) nystatin A1 is the major metabolic shunt product under glycosylation deficiency. Following *nysGtf* disruption, nystatin A1 accumulated to 58.6% of total derivatives. This indicates that blocking C-35 glycosylation diverts precursor metabolites (10-deoxynystatin) away from A3/polyfungin B pathways and toward the non-glycosylated A1, identifying A1 as the default end product when glycosylation is impaired. There are three possible underlying mechanisms for these results. First of all, substrate competition and enzyme specificity, the glycosyltransferase encoded by *nysGtf* may have higher affinity for the A3 precursor than for polyfungin B intermediates, explaining the preferential boost in A3 during overexpression. Secondly, metabolic flux redistribution, disruption of C-35 glycosylation eliminates branching toward A3/polyfungin B, forcing all precursors into the A1 biosynthesis pathway. Conversely, overexpression of *nysGtf* enhances flux through the glycosylation branch but is limited by upstream precursor availability for polyfungin B. In the end, regulatory constraints for polyfungin B, polyfungin B biosynthesis may require additional rate-limiting factors (cosubstrates, accessory enzymes, or transcriptional regulation) that are not alleviated by *nysGtf* overexpression alone, preventing its yield from exceeding parental levels [32,33,34,35]. The validated functions of *nysL* and *nysGtf* facilitate their heterologous expression in chassis organisms, enabling novel production of nystatin analogs. This lays the groundwork for combinatorial biosynthesis, where modular assembly of functional domains could generate antibiotics with novel pharmacological profiles [36].

The elucidation of the gene functions involved in the biosynthetic pathways of nystatin A3 and polyfungin B, along with the construction of high-producing polyfungin B strains, expands our understanding of polyene macrolide antibiotic biosynthesis. This knowledge provides a theoretical foundation for engineering *S. noursei* 72-22-1 to optimize metabolic product yields through genetic modification.

## 4. Materials and Methods

### 4.1. Strains, Plasmids, and Culture Conditions

*Streptomyces noursei* 72-22-1 and *Escherichia coli* DH-5α (DE3) were preserved in the laboratory. *E. coli* ET12567 (pUZ8002) was generously provided by Dr. Huanzhang Xia’s laboratory.

The plasmids used in this experiment are shown in Table 4. *E.coli* strains were cultured on Luria–Bertani (LB) agar or in LB liquid medium containing appropriate antibiotics for selection at 37 °C(Qingdao Hope, Qingdao, China) [37]. Gause’s synthetic agar (20 g/L soluble starch, 1 g/L KNO_3_, 0.5 g/L K_2_HPO_4_, 0.5 g/L MgSO_4_·7H_2_O, 0.5 g/L NaCl, 0.01 g/L FeSO_4_·7H_2_O, and 20 g/L agar, pH 7.2) (Qingdao Hope, Qingdao, China) was used for sporulation of *S. noursei* and its mutant derivatives [14,20]. Seed medium (g/L): glucose 20, peptone 5, yeast extract 5, NaCl 2, pH 7.2. Fermentation production medium (g/L): glucose 55, peanut meal 25, peptone 5, CaCO_3_ 5, pH 7.5. All *Streptomyces* strains were cultured using two-stage fermentation at 220 rpm and 28 °C. After 24 h of incubation at 28 °C seed cultures were used to inoculate fermentation medium at 10% (*v*/*v*), which were then incubated for 86 h [4,20].

### 4.2. Genetic Procedures

Plasmid DNA from *E. coli* was extracted using the AxyPrepTM Plasmid DNA Extraction Kit (Axygen Biosciences, Union City, CA, USA). Genomic DNA from *Streptomyces* was extracted according to the instructions in the TIANamp Bacterial Genomic DNA Kit (Tiangen Biotech, Beijing, China). Preparation of competent *E. coli* cells and DNA transformation were conducted using the calcium chloride (CaCl_2_) method, following protocols described in Molecular Cloning [39] (Table 5).

### 4.3. Conjugation Transfer Experiment

Five milliliters of sterile water were added to a sporulated *S. noursei* slant culture, and spores were scraped using an inoculating loop. The spore suspension was mixed by inversion for 30 min and filtered through absorbent cotton to obtain a monospore suspension, which was counted microscopically. Spores were collected by centrifugation at 3500 rpm for 10 min, washed twice with 10 mL YT medium, and resuspended to a target concentration of 10^8^ CFU/mL. The suspension was heat-shocked at 50 °C for 10 min, germinated at 37 °C for 3 h, and cooled to room temperature for use as the recipient strain.

*E. coli* ET12567 (pUZ8002) containing the target plasmid was inoculated at 2% (*v*/*v*) into 3 mL LB medium supplemented with 25 μg/mL chloramphenicol, 25 μg/mL kanamycin, and 50 μg/mL apramycin. The culture was incubated at 37 °C for 12 h, then inoculated at 2% (*v*/*v*) into 20 mL LB medium containing the same antibiotics and grown at 37 °C for 4–5 h to an OD600 of 0.5–0.6 [40,41]. Cells were harvested by centrifugation at 3000 rpm for 10 min, washed twice with LB medium, and resuspended in 5 mL LB medium as the donor strain.

Equal volumes (500 µL) of recipient and donor strains cultures were mixed, centrifuged at 3000 rpm for 10 min, and resuspended in the remaining liquid. The mixture was plated on MS agar and incubated at 28 °C for 20 h [42]. Plates were overlaid with 1 mL sterile water containing 20 µL nalidixic acid (50 mg/mL) and 20 µL apramycin (75 mg/mL) and air-dried in a biosafety cabinet. Conjugates were cultured at 28 °C for seven days, and single colonies were transferred to Gause’s synthetic agar containing 75 µg/mL apramycin and 50 µg/mL nalidixic acid for further selection. Conjugates potentially harboring the target plasmid were isolated.

### 4.4. Construction of Disruption and Overexpression Plasmids for nysL and nysGtf

The upstream and downstream homologous arms of *nysL* were amplified from the genomic DNA of *S. noursei* 72-22-1 using primers L-UF/L-UR and L-DF/L-DR, respectively. The amplified fragments were ligated into the temperature-sensitive plasmid pKC1139 using the Trelief^®^ SoSoo Cloning Kit Ver.2 (TSINGKE Biotechnology, Beijing, China) to construct the nysL knockout plasmid pKC1139-LUD. Similarly, the homologous arms of *nysGtf* were amplified with primers G-UF/G-UR and G-DF/G-DR and cloned into pKC1139 to generate the *nysGtf* knockout plasmid pKC1139-GUD. For overexpression plasmid construction, the full-length *nysL* and *nysGtf* were amplified using primers L-F/L-R and G-F/G-R, respectively. These genes were subsequently cloned into the integrative plasmid pSET152 via the same cloning kit, yielding the overexpression plasmids pSET152-*nysL* and pSET152-*nysGtf*. The composition of the ligation reaction system is detailed in Table S2. Single and double recombinants were selected and screened as described by Ni [43].

### 4.5. Extraction and Purification of Fermentation Products

Fermentation broth was centrifuged at 3500 rpm for 5 min to collect wet mycelia. The biomass was extracted with 98% ethanol at three times its weight (*w*/*v*) with stirring for 40 min, followed by centrifugation at 3500 rpm for 10 min. The residue was re-extracted twice with 98% ethanol. The combined extracts were concentrated under reduced pressure (93.3 kPa) to 15% of the original volume.

The concentrate was stored at 4 °C for 24 h to induce crystallization. The crystals were separated by centrifugation, washed with ethyl acetate and saline, and then washed again with ethyl acetate before being dried. Purified fermentation products were stored in light-protected conditions.

### 4.6. HPLC and HPLC-MS Analysis of Fermentation Products

Fifty milligrams of the purified fermentation product were dissolved in 5 mL methanol and analyzed by HPLC (High-Performance Liquid Chromatography). The HPLC conditions were as follows: Shimadzu C18 Superb column (250 × 4.6 mm, 5 µm), mobile phase consisting of methanol:acetonitrile:0.05 M sodium acetate buffer (pH 4.0) = 37:26:37 (*v*/*v*/*v*), flow rate of 1 mL/min, injection volume of 10 µL, detection wavelength of 305 nm, and column temperature of 30 °C [44]. A purified fermentation product (25 mg) was dissolved in 5 mL of methanol and analyzed using HPLC. The HPLC conditions were as follows: column, Unitary C18 (250 × 4.6 mm, 5 µm); mobile phase, methanol:water = 70:30 (*v*:*v*; flow rate, 1 mL/min; injection volume, 10 µL; detection wavelength, 305 nm; and column temperature, 30 °C. The MS conditions included an electrospray ionization (ESI) source in full-scan mode, with a cone voltage of 15 V and a capillary voltage of 0.8 kV [44,45]. For quantification: authentic commercial standards of nystatin A1, nystatin A3 and polyfungin B were used to build external-standard calibration curves (peak area versus concentration, mg/mL), purchased from Zhejiang Zhenyuan, Shaoxing, China. For 10-deoxynystatin, no commercial authentic standard was available. Since all nystatin-related compounds share the identical tetraene chromophore, they exhibit highly similar molar extinction coefficients at 305 nm. Therefore, nystatin A1 standard was applied as reference, with relative-response-factor = 1, to calculate 10-deoxynystatin concentration from its HPLC peak area. All quantifications were performed from at least three biological replicate fermentations.

### 4.7. Identification of Fermentation Products by ESI-MS and NMR

Fermentation products were identified by ESI-MS and NMR analysis under standard conditions. ^1^H and ^13^C NMR spectra were recorded using a Brüker AVANCE III 600 instrument (Bruker Biospin AG, Fällanden, Switzerland). ^1^H NMR spectra were obtained in CDCl_3_ and DMSO-*d*_6_, with tetramethylsilane (TMS) as an internal standard. Mass spectra were recorded in ESI mode using an Agilent 1200 LC-MS (Agilent Technologies, Santa Clara, CA, USA).

### 4.8. Statistical Analysis

All experiments were performed with at least three biological replicates. Statistical analysis was conducted using SPSS Statistics 20.0 software. Statistical significance was determined using Student’s *t*-test for pairwise comparisons, with a *p*-value < 0.05 considered significant.

## 5. Conclusions

In this work, we performed targeted gene disruption, complementation and overexpression to functionally characterize two tailoring enzymes NysL (P450 monooxygenase) and NysGtf (glycosyltransferase) in *Streptomyces noursei* 72-22-1. We experimentally confirmed that NysL catalyzes C-10 hydroxylation and NysGtf mediates C-35 digitoxose glycosylation during nystatin biosynthesis.

Compared with previous studies on nystatin biosynthesis, this study provides several novel insights. Earlier reports characterized NysL activity but lacked quantitative metabolite profiling for full nystatin derivative panels; although its close homologue SnNysSV from reference *S. noursei* has been biochemically characterized in vitro [19], the in vivo function of the C-35 glycosyltransferase NysGtf from the industrial strain *S. noursei* 72-22-1 had remained only bioinformatically predicted before this work. We clarified the substrate preference of NysGtf toward different pathway intermediates and revealed metabolic branching flux redistribution upon glycosylation blockage. In addition, we successfully obtained and structurally verified the key intermediate 10-deoxynystatin, which was difficult to acquire in previous strain systems.

For practical implementation, our genetic-engineering strategies offer feasible routes for strain improvement for industrial nystatin production. Knockout of *nysL* enables high accumulation of polyfungin B, which possesses superior antifungal activity compared with nystatin A1 and A3, supporting construction of high-polyfungin-B industrial strains. Overexpression of *nysGtf* can specifically boost nystatin A3 titers. The engineered mutants also supply abundant biological sources for rare polyene intermediates, which can serve as starting materials for semi-synthetic modification to develop less-toxic polyene antifungal derivatives. In future work, combinatorial manipulation of *nysL* and *nysGtf*, together with precursor-supply optimization, could further redirect metabolic flux toward desired high-activity nystatin derivatives, facilitating industrial manufacture of improved polyene antifungal agents.

## Figures and Tables

**Table 1:** ^13^C NMR chemical-shift assignments for product I, which was identified as nystatin A1. Spectra were acquired in DMSO-*d*_6_ solvent; chemical shifts are given in parts per million (ppm).

Position	Chemical Shift (ppm)	Position	Chemical Shift (ppm)
	^13^C NMR		^13^C NMR
1	170.91	25	133.4
2	43.07	26	131.6
3	66.41	27	131.55
4	44.86	28	32.31
5	69.91	29	32.16
6	44.78	30	134.73
7	70.85	31	131.47
8	34.89	32	129.71
9	28.96	33	135.9
10	73.85	34	40.93
11	71.1	Me34	17.37
12	42.61	35	76.46
13	97.64	36	40.93
14	-	Me36	12.56
15	66.03	37	70.69
16	58.34	Me37	17.09
17	66.23	Mycosamine moiety1′	97.82
18	37.89	2′	68.43
19	75.66	3′	56.38
20	134.73	4′	71.1
21	131.72	5′	73.26
22	133.4	6′	18.37
23	133.4	COOH	179.19
24	129.92		

**Table 2:** ^1^H and ^13^C NMR chemical-shift assignments for product II (identified as nystatin A3). Spectra were acquired in DMSO-*d*_6_ solvent; chemical shifts are reported in parts per million (ppm).

Position	Chemical Shift (ppm)		Position	Chemical Shift (ppm)		Position	Chemical Shift (ppm)	
	^13^C NMR	^1^H NMR		^13^C NMR	^1^H NMR		^13^C NMR	^1^H NMR
1	170.36	-	19	76.34	4.36	36	41.33	4.72
2	42.08	2.35	20	134.85	-	Me36	11.34	0.92
3	65.43	4.02	21	131.50	-	37	69.12	4.48
4	44.04	1.47	22	131.63	-	Me37	17.20	1.32
5	70.11	4.02	23	134.44	-	COOMe	174.27	-
6	43.61	-	24	127.87	-	1′	97.83	4.72
7	69.31	-	25	132.47	3.97	2′	68.03	-
8	33.82	1.40–1.52	26	130.91	-	3′	55.11	-
9	30.06	-	27	130.20	-	4′	71.65	-
10	74.17	3.24	28	32.47	2.16	5′	16.98	-
11	70.28	-	29	32.02	2.16	6′	72.80	1.16
12	37.89	-	30	134.77	5.53	1″	97.83	4.78
13	97.31	-	31	129.64	5.92	2″	37.45	-
14	44.71	-	32	127.87	5.96	3″	67.89	-
15	65.79	4.09	33	138.07	5.49	4″	72.29	-
16	56.03	1.93	34	36.96	2.03	5″	72.63	-
17	65.28	3.84	Me34	17.79	1.11	6″	17.74	0.99
18	35.39	1.76	35	78.03	3.17			

**Table 3:** ^1^H and ^13^C NMR chemical-shift assignments for product IV (identified as polyfungin B). Spectra were acquired in DMSO-*d*_6_ solvent; chemical shifts are reported in parts per million (ppm).

Position	Chemical Shift (ppm)		Position	Chemical Shift (ppm)		Position	Chemical Shift (ppm)	
	^13^C NMR	^1^H NMR		^13^C NMR	^1^H NMR		^13^C NMR	^1^H NMR
1	170.39	-	19	76.34	4.36	36	41.35	-
2	42.06	2.39	20	134.84	5.7	Me36	11.34	0.96
3	65.43	3.95	21	131.52	-	37	69.13	4.48
4	44.05	1.49	22	131.63	-	Me37	17.26	1.36
5	70.11	-	23	134.43	-	COOMe	174.24	-
6	43.62	1.47	24	127.88	-	1′	97.83	4.71
7	69.12	-	25	132.49	-	2′	67.97	3.76
8	41.65	1.46	26	130.92	-	3′	55.10	2.85
9	17.44	-	27	130.21	-	4′	71.62	-
10	38.24	1.23	28	32.46	2.16	5′	16.98	-
11	72.36	-	29	32.02	2.16	6′	72.83	1.15
12	44.62	-	30	134.74	5.54	1″	97.84	4.77
13	97.32	-	31	129.63	5.91	2″	37.46	1.87
14	44.73	1.23, 1.87	32	127.87	5.96	3″	67.85	-
15	65.80	4.03	33	138.06	5.47	4″	72.31	-
16	56.03	1.93–1.87	34	36.95	-	5″	72.60	-
17	65.29	3.89	Me34	17.79	1.11	6″	17.72	0.96
18	35.38	1.76	35	78.03	3.16			

**Table 4:** Plasmids used in this study.

Plasmids	Description/Derivation	Reference
pKC1139	*E. coli*-*Streptomyces*, shuttle plasmid, temperature sensitive type, Am^R^, *ori_SG5_*, *orT*	Honorgene, [38]
pKC1139-LUD	*nysL* blocking plasmid, pKC1139 with upstream and downstream homologous arms of *nysL*, Am^R^	constructed by our laboratory
pSET152-KT	*E. coli*-*Streptomyces*, shuttle plasmid, site-specific integration, Am^R^, *ori_pUC18_*, *oriT*, φ*intC31*, P_kasop*_, *T*0	Genomics
pSET152-nysL	pSET152-KTwith *nysL*, Am^R^	constructed by our laboratory
pKC1139-GUD	*nysGtf* blocking plasmid, pKC1139 with upstream and downstream homologous arms of *nysGtf*, Am^R^	constructed by our laboratory
pSET152-nysGtf	pSET152-KT with *nysGtf*, Am^R^	constructed by our laboratory

**Table 5:** Oligonucleotide primers used in this study.

Name	Sequence (5′→3′)
L-UF	CAAGCTTGGGCTGCAGGTCGACTCTAGAGCCGACCGGGACTTCT
L-UR	CCCCTGCTGCCGCCTACACCCTGCTCTT
L-DF	GGTGTAGGCGGCAGCAGGGGGCTCAGG
L-DR	GGATCCGCGGCCGCGCGCGATATCGAATGCGATGCGGATGCTCAAG
NL-5	ACGCTGATCCCCGAGTT
NL-2	GGCCGGACAAGCCGC
NL-7	AAGAGCAGGGTGTAGGCG
NL-8	TCTTCGACAGCAAGTCCC
L-R	GGACTAGTCCATAAGAATGCGGCCGCATGAGCACACCCACCGCG
L-F	ACCGGATCTGCAGCGGATCCTCACCAGGTGACGGACAGCTGC
G-UF	CAAGCTTGGGCTGCAGGTCGACTCTAGACGCGTACCGGACCGAG
G-UR	GTGTCGGCGAACGACGGACTGGTGATCAGC
G-DF	CCAGTCCGTCGTTCGCCGACACCTTCGCG
G-DR	TATCGCGCGCGGCCGCGGATCCTCTAGAGATCGACCCGCAGCAGG
NG-5	CGCTTTCGCCAGTCGTTC
NG-6	GGTCGTTCTGCTGCTCAAC
NG-7	TGCCCAGTTATGCCGACT
NG-8	AGGACGAGTGCCTTCACG
G-F	GACTAGTCCATAAGAATGCGGCCGCATGCGTGTCCTGCTGATCAC′
G-R	ACCGGATCTGCAgcGGATCCTCATGTGCCCGCTCCC

## Data Availability

The original contributions presented in this study are included in the article/Supplementary Materials. Further inquiries can be directed to the corresponding author(s).

## Supplementary Materials

The following supporting information can be downloaded at: https://www.mdpi.com/article/10.3390/ijms27219572/s1.
