# Supplementary material for: Optimizing Nystatin Biosynthesis Through Targeted Manipulation of nysL and nysGtf in Streptomyces noursei
Source: Int J Mol Sci. 2026 Oct 26;27(21):9572. doi: 10.3390/ijms27219572 (PMC13763215; doi:10.3390/ijms27219572)

Supplementary material

Optimizing nystatin biosynthesis through targeted manipulation of *nysL* and *nysGtf* in *Streptomyces noursei*

Table S1 Composition of High- PCR reaction fidelity system

Composition	Final concentration	Volume（μl）
2×PrimeSTAR GC Buffer (Mg^{2+} plus)	1×	25.0
dNTP Mixture (2.5 mM each)	0.4 mmol·L^{-1}	4.0
Primer 1	0.25 μmol·L^{-1}	4.0
Primer 2	0.25 mmol·L^{-1}	4.0
Genomi DNA		2.0
PrimeSTAR HS DNA Polymerase (2.5 U/μl)	1.25 U	0.5
ddH_2O		10.5
Total volume（μl）		50.0

PCR was performed using the following thermal cycling conditions: an initial denaturation step at 95°C for 5 minutes, followed by 45 seconds of denaturation at 95°C, annealing at the primer-specific Tm minus 5–10°C for 45 seconds, and an extension at 72°C for 2 minutes, with a final extension step at 72°C for 10 minutes.

Table S2 Composition of ligation reaction system

	Double fragment insertion	Single fragment insertion
Linearized vecctors	1μl	1μl
Insertion fragment 1	2μl	2μl
Insertion fragment 2	2μl	-
2×SoSoo Mix	5μl	5μl
ddH_2O	0μl	2μl

Table S3 Amino acid sequence alignment of NysGtf and SnNysSV

```
nysGtf             1 MRVLLITSPSATHFLPMVPLAWALRAAGHDVLVAGQPDVLDAVAGAGLNA     50
                     ||||||||||||||||||||||||||||||||||||||||||||||||||
SnNysSV            1 MRVLLITSPSATHFLPMVPLAWALRAAGHDVLVAGQPDVLDAVAGAGLNA     50

nysGtf            51 VPTGRRAGIDDAMRELLFRPGLRPFECVGRWTPEMLAAFPPVWQRHSADV    100
                     ||||||||||||||||||||||||||||||||||||||||||||||||||
SnNysSV           51 VPTGRRAGIDDAMRELLFRPGLRPFECVGRWTPEMLAAFPPVWQRHSADV    100

nysGtf           101 LPRYLDLARAHRPDLIVCDVMEFNAPVVGAHVGVPVVRHRYGVDPLLGGV    150
                     ||||||||||||||||||||||||||||||.:|:||||||||||||||||
SnNysSV          101 LPRYLDLARAHRPDLIVCDVMEFNAPVVGAQLGIPVVRHRYGVDPLLGGV    150

nysGtf           151 GPAARAALRSLHEELGLAELPEPTAVIDPCPADLQLPGVAPGLPMRYVPY    200
                     |||||||||||||||||||||||||||||||||||||||||||.||||||
SnNysSV          151 GPAARAALRSLHEELGLAELPEPTAVIDPCPADLQLPGVAPGLAMRYVPY    200

nysGtf           201 NGNGGLPDWLRAERRAGAPPRRVLVSLGSHTLALNGVPLLRGILAAAGDG    250
                     ||||.|||||||||||||||||||||||||||||||||||||||||||||
SnNysSV          201 NGNGVLPDWLRAERRAGAPPRRVLVSLGSHTLALNGVPLLRGILAAAGDG    250

nysGtf           251 SSGVEVLATVPEAYRAELGPVPDTVRLIDPLPLHLIAGDCAVVVHHGGAG    300
                     |||||||||||||||||||||||||||||||||||||||||.||||||||
SnNysSV          251 SSGVEVLATVPEAYRAELGPVPDTVRLIDPLPLHLIAGDCAAVVHHGGAG    300

nysGtf           301 TGMTVAGLGVPQLVLPQFADTFAFAERLAAVGAGMAVDTVEQQNDPQFLR    350
                     ||||||||||||||||||||||||||||||||||:|||||||||||:|||
SnNysSV          301 TGMTVAGLGVPQLVLPQFADTFAFAERLAAVGAGVAVDTVEQQNDPRFLR    350

nysGtf           351 KALQALLGEPSYGHAAERLRHTMEAMPPPAALVPQLARIAAGAGT    395
                     |||..|||||||||||||||||||||||||:|||||||||.
SnNysSV          351 KALGTLLGEPSYGHAAERLRHTMEAMPPPAALVPELARIAAGAGA    395
```

Figure S1 Construction of the *nysGtf* Gene Disruption Strain. A.Workflow for constructing pKC1139-GUD.B.Restriction enzyme analysis of pKC1139-GUD.M:DL15000 DNA Marker; Lane1:pKC1139-GUD(*Hind III/EcoR I*) C. PCR product analysis of the *nysGtf* disruption strain. Lane1. PCR product using upstream verification primers for the double-crossover strain; Lane2. PCR product using downstream verification primers for the double-crossover strain; Lane3.PCR product using upstream verification primers for the wild-type strain; Lane4. PCR product using downstream verification primers for the wild-type strain; M: DL15,000 DNA Marker

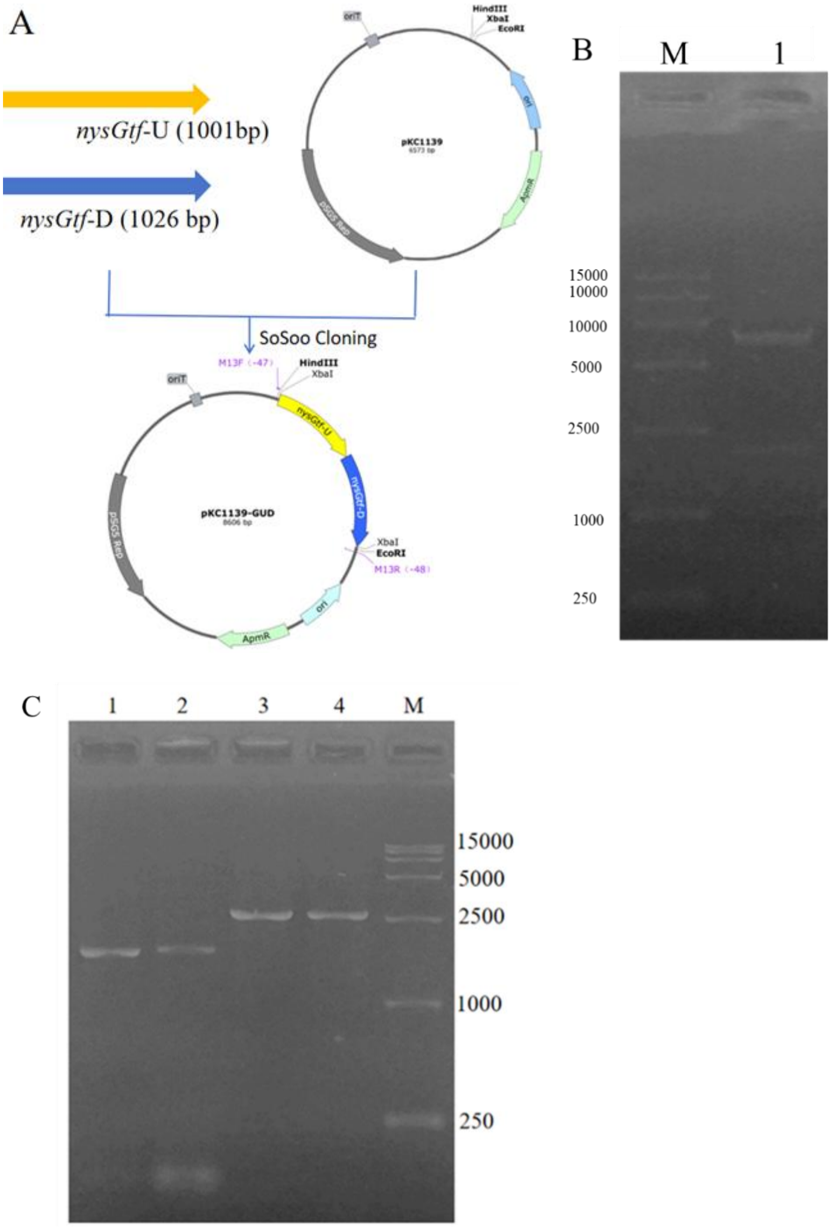

Figure S2 MS analysis results of product III

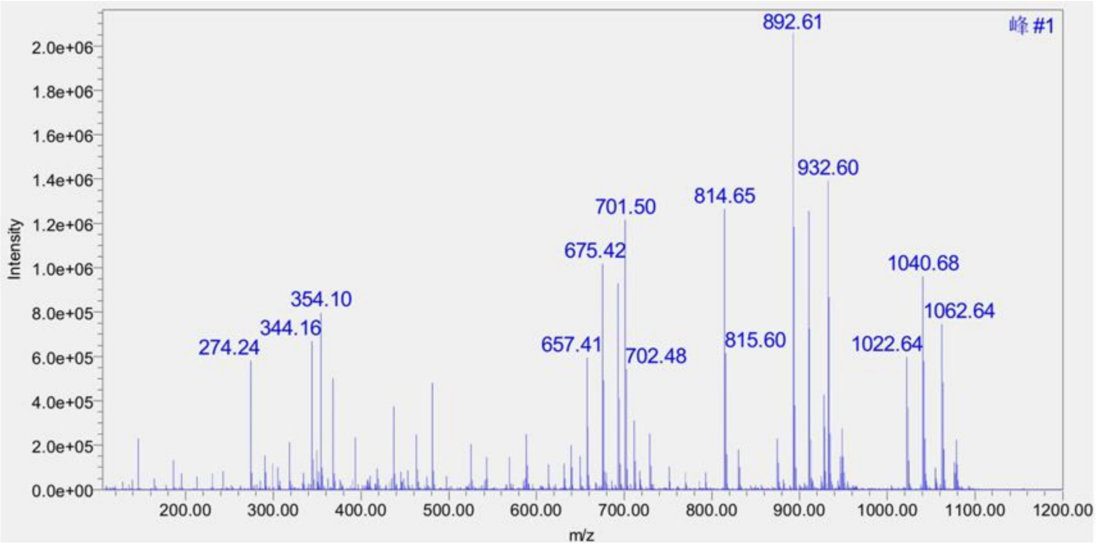

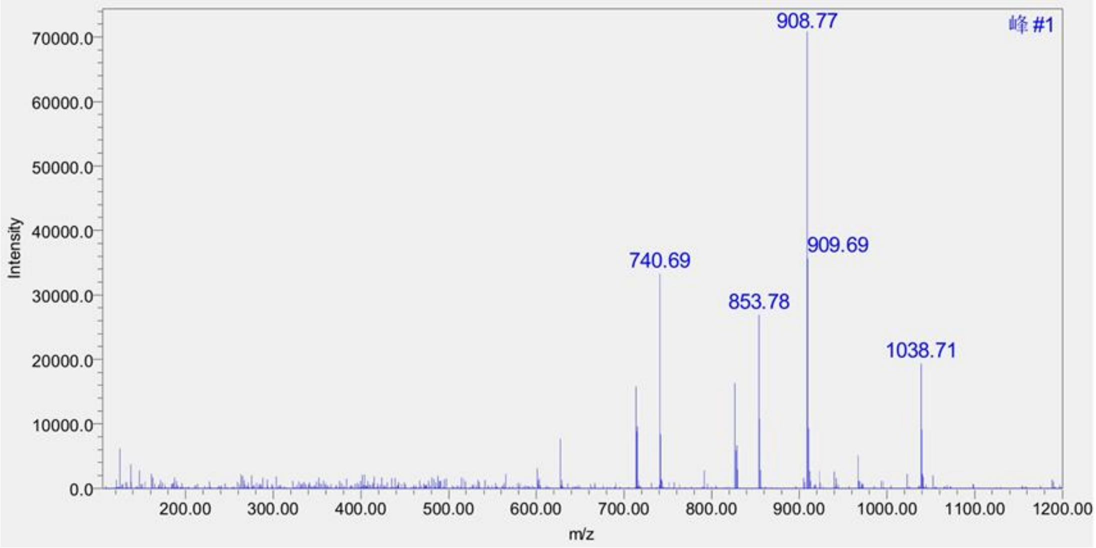

Supplement: Supplementary file 1 [file ijms-27-09572-s001.zip › ijms-4599778-supplementary.pdf]
